# Multifocal alopecia of the scalp, axillae, and body

**DOI:** 10.1016/j.jdcr.2024.05.005

**Published:** 2024-05-16

**Authors:** Fabiola Moreno Echevarria, Claudia S. Roldan, Alyce Anderson, Jennifer L. Shastry

**Affiliations:** Department of Dermatology, Northwestern Feinberg School of Medicine, Chicago, Illinois

**Keywords:** alopecia, Graham-Little-Piccardi-Lasseur syndrome, lichen planopilaris

## Case summary

**Fig 1:**
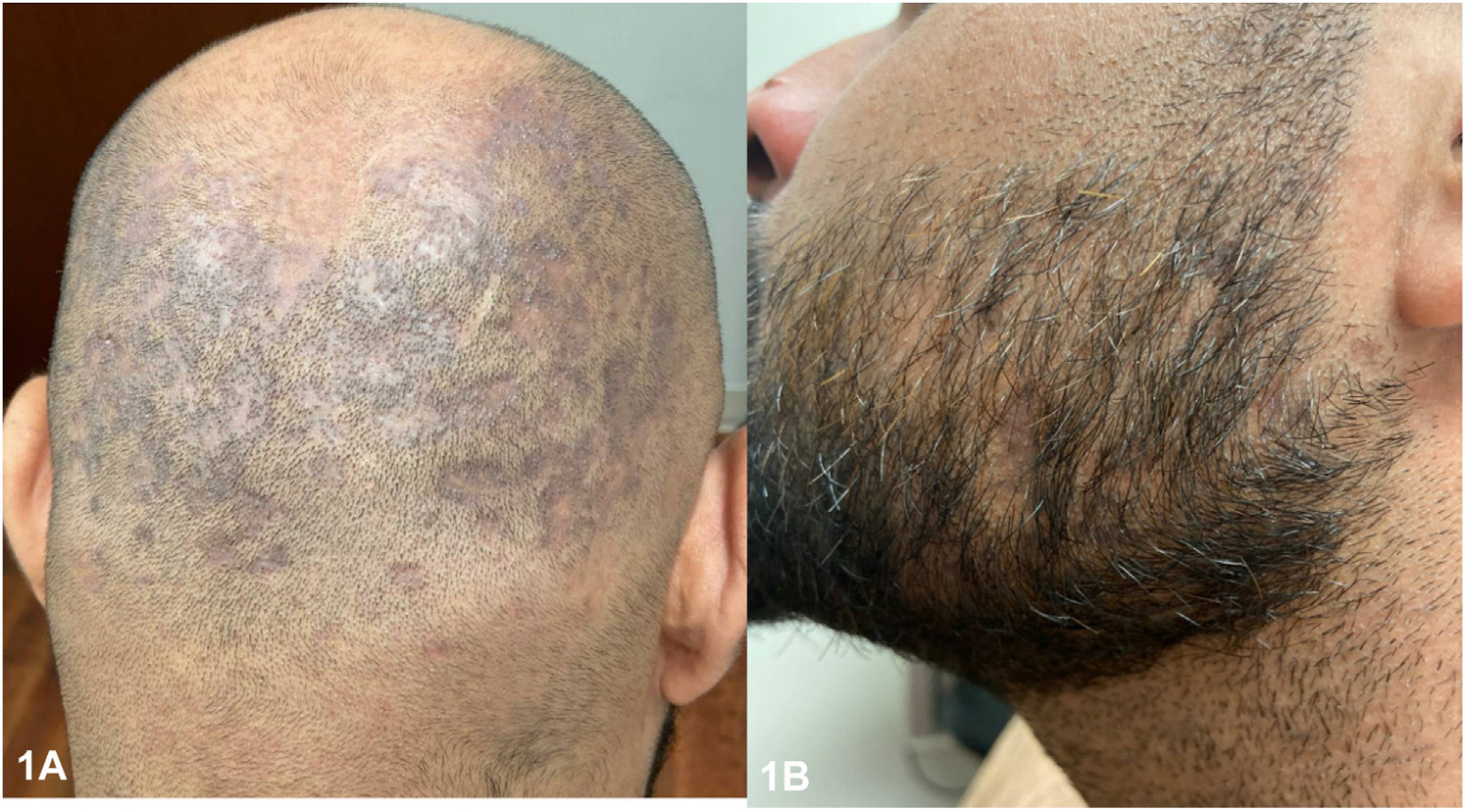
▪▪▪

**Fig 2:**
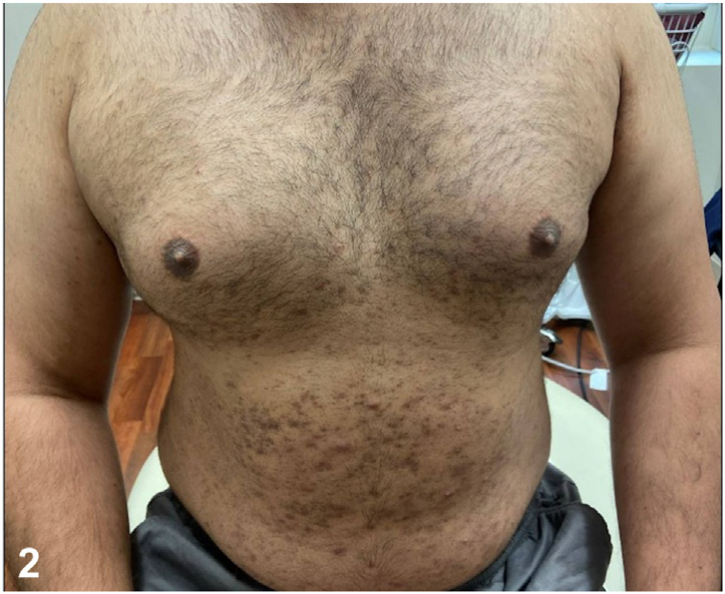
▪▪▪

**Fig 3:**
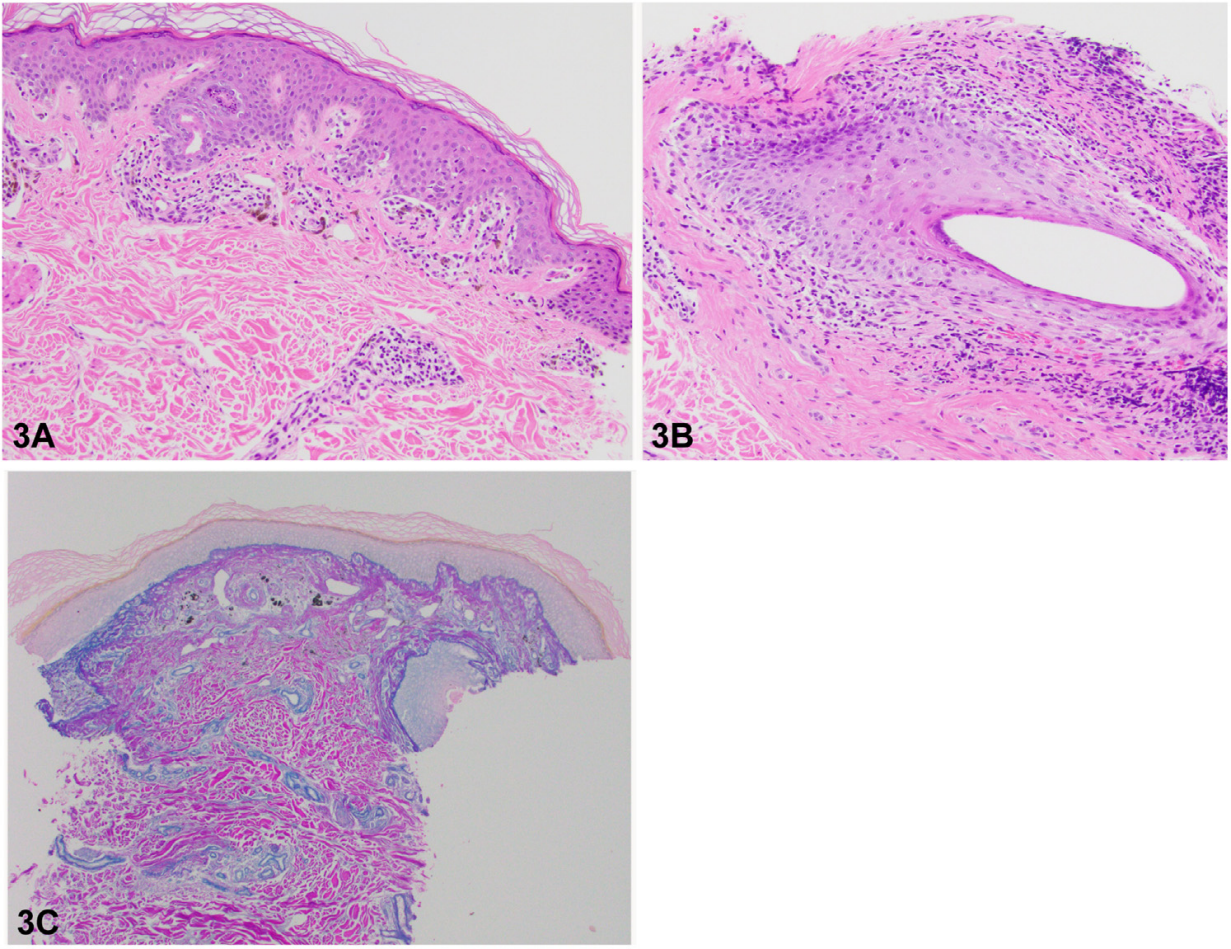
▪▪▪

A 29-year-old man presented with a year of nonpruritic eruption with associated hair loss. Scalp and beard examination demonstrated scattered thin pink to violaceous papules coalescing into plaques with patchy scarring alopecia (Fig 1). On the body, there were eruptions of follicularly based keratotic violaceous papules (Fig 2), whereas on the suprapubic skin and bilateral axillae there were brown to violaceous thin papules and plaques with nonscarring alopecia. Conchal bowls were clear. He denied systemic symptoms, arthralgias, or family history of autoimmune disease. Antinuclear antibody and treponemal stain were negative. A biopsy supported the diagnosis (Fig 3).

**Question 1: What is the most likely diagnosis?**

A. Pseudopelade of Brocq
B. Frontal fibrosing alopecia
C. Alopecia areata
D. Graham-Little-Piccardi-Lasseur syndrome (GLPLS)
E. Discoid lupus erythematosus (DLE)

**Answers:**

A. Pseudopelade of Brocq – Incorrect. Pseudopelade of Brocq is a rare cicatricial alopecia presenting with discrete, asymmetric, and nonscaly patches of alopecia. No significant inflammation is seen on histopathology.
B. Frontal fibrosing alopecia – Incorrect. Frontal fibrosing alopecia, a variant of lichen planopilaris, is a form of cicatricial alopecia clinically characterized by band-like alopecia of the frontal scalp with associated eyebrow thinning.
C. Alopecia areata – Incorrect. Alopecia areata is a nonscarring alopecia with peribulbar lymphocytic infiltrate on histopathology.
D. GLPLS – Correct. A variant of lichen planopilaris, GLPLS classically presents as a triad of multifocal cicatricial alopecia of the scalp, nonscarring alopecia of the axillae and/or groin and keratotic follicular papules of the trunk and extremities. Histologic findings can include a perifollicular lymphocytic infiltrate with necrotic keratinocytes, perifollicular fibroplasia, and follicular dilatation with plugging.^1^, ^2^, ^3^
E. DLE – Incorrect. DLE is a clinical and histologic mimicker of GLPLS. The presence of keratotic follicular papules and nonscarring alopecia on the trunk, lack of conchal bowl involvement, histopathologic findings, and negative antinuclear antibody all contributed to favoring a diagnosis of GLPLS over DLE in this case. While both GLPLS and DLE may show hyperkeratosis, follicular dilatation and plugging on histopathologic examination, basement membrane zone thickening and mucin deposition are features of DLE that were not seen in this case.^4^

**Question 2: Which of the following would be considered first-line in treatment of this condition?**

A. Topical steroids
B. Tofacitinib
C. Doxycycline
D. Hydroxychloroquine
E. Minoxidil

**Answers:**

A. Topical steroids – Correct. Both topical and intralesional steroids are considered first-line therapy for lichen planopilaris (LPP) and its variants including GLPLS. Steroids have been shown to reduce clinical signs of inflammation and halt progression of cicatricial alopecia. They can be used alone or in combination with other agents.^5^
B. Tofacitinib – Incorrect. Janus kinase upregulation has been thought to play a role in LPP and its variants such as GLPLS. The use of oral tofacitinib has been reported in recalcitrant cases of GLPLS, however is not considered a first-line therapy.^1^^,^^5^
C. Doxycycline – Incorrect. Although doxycycline, an oral antibiotic with antiinflammatory properties, is commonly used in some cicatricial alopecias such as frontal fibrosing alopecia, it is not considered first-line in treatment of GLPLS.
D. Hydroxychloroquine – Incorrect. Hydroxychloroquine, widely used in the treatment of autoimmune conditions, has been reported as a treatment for cases of GLPLS refractory to first-line treatment with topical and/or intralesional steroids. Hydroxychloroquine has been shown effective for controlling symptoms, significantly reducing disease severity, and halting progression of LPP.^5^
E. Minoxidil – Incorrect. Minoxidil is used to treat other etiologies of alopecia such as androgenic or alopecia areata, but it is not typically used as first-line treatment of LPP and its variants.

**Question 3: Which of the following is true regarding this condition?**

A. Complete remission of disease is often achieved with use of topical steroids
B. Anagen pull test is typically negative
C. Histologic findings are variable depending on the stage of development
D. Lesions are typically nontender and nonpruritic
E. It has been most frequently described in young males

**Answers:**

A. Complete remission of disease is often achieved with use of topical steroids – Incorrect. Treatment of cicatricial alopecia is difficult. Treatment options that have been reported to provide symptomatic relief and slow disease progression include topical steroids, hydroxychloroquine, Janus kinase inhibitors, systemic retinoids, and peroxisome proliferator activated receptor γ agonists such as pioglitazone. Although improvement may be seen with treatment, complete resolution is rare.^1^^,^^5^
B. Anagen pull test is typically negative – Incorrect. Those affected with GLPLS have relatively weak stabilization of hair follicles. This commonly results in a positive anagen hair pull test.
C. Histologic findings are variable depending on the stage of development – Correct. The histopathology of GLPLS has been described as similar to that of classical LPP, with early lesions showing perifollicular lymphocytic infiltrates and vacuolar changes of the outer root sheath, whereas evolved lesions exhibit signs of cicatricial alopecia such as concentric perifollicular fibrosis and follicular scars. As the condition progresses, only cicatricial alopecia patches are typically found. It important to note that these characteristics can vary depending on the biopsy location given the different components of the GLPLS triad.^3^
D. Lesions are typically nontender and nonpruritic – Incorrect. Pruritus is the most frequent complaint of those presenting with GLPLS.^1^ Asymptomatic hair loss has been observed in minority of cases, as observed in the case we present.^1^
E. It has been most frequently described in young males – Incorrect. Although we present a case of a young male with GLPLS, this disease is more common in postmenopausal females, with few reported cases affecting young males.^2^

## Conflicts of interest

None disclosed.
